# The trends of blood culture contamination and utilization rates in an LMIC tertiary care center from 2010 to 2022: a call for diagnostic stewardship?

**DOI:** 10.1017/ash.2024.479

**Published:** 2025-01-27

**Authors:** Lama Saleh, Amanda Chamieh, Rima El Basst, Eid Azar

**Affiliations:** 1 Department of Infectious Diseases, Saint George Hospital University Medical Center, Beirut, Lebanon; 2 Department of Infection Prevention and Control and Antimicrobial Stewardship, Saint George Hospital University Medical Center, Beirut, Lebanon

## Abstract

**Objective:**

At Saint George Hospital University Medical Center in Beirut, Lebanon, we determine (1) annual blood culture (BC) contamination (BCC) and utilization (BCU) rates vs international benchmarks, (2) identify blood culture contaminants, (3) bloodstream infections episodes in patients with and without COVID-19 after the pandemic onset, and (4) any epidemiologic trends in BCC and BCU.

**Design:**

Retrospective observational study.

**Setting:**

Private tertiary referral center, from January 1, 2010, to December 31, 2022.

**Methods:**

We define a contaminated BC as the growth of a typical contaminant/skin flora in 1-2/4 BC bottles. We calculate BCC rates as a percentage of the contaminated BC/total BC during the period and BCU rates as the number of BC/1000 patient days (PD).

**Results:**

The average BCU rate of 85.9/1000 PD in 2010–2019 increased to 106.6/1000 PD in 2020–2022. On average, patients with COVID-19 had a higher BCU rate of 185.9/1000 PD, corresponding to an additional 100 blood cultures/1000 PD. The average BCC rate was 7%, ranging from 6% in 2010–2019 to 8% in 2020–2022. We observed the highest BCC rate of 9% in patients with COVID-19, likely due to the higher BCU. The most frequently isolated contaminants were coagulase-negative Staphylococcus (96%), of which 65% were Staphylococcus epidermidis.

**Conclusion:**

We saw a multifactorial, persistently elevated rate of BCC over 13 years as unaffected by strict infection control practices. We think that further research targeting a standardized, low BCU rather than inevitable BCC while advocating for diagnostic stewardship of low-middle-income countries is essential, especially where the lack of appropriate resource allocation and awareness are problematic.

## Introduction

For as long as we can remember, the diagnosis of bloodstream infections (BSIs) has relied on blood cultures.^1^ Primary BSIs occur in the presence of central lines,^2^ while secondary BSIs are a manifestation of a distant infection.^3^ Blood cultures are an essential component of sepsis bundles and play a role in narrowing empirical therapy and limiting exposure to broad-spectrum antibiotics, and eventually, the emergence of resistant strains.^4^ In addition, pathogen identification, classification as community or healthcare-associated, and their antimicrobial susceptibility patterns have an epidemiologically important role and allow a better selection of empirical coverage.^5^

Similarly, the timing of blood culture collection can alter treatment because delaying sampling is associated with a 20% reduction in pathogen detection if cultures are collected while on antibiotic therapy.^6^ If collected in a proper and timely manner, blood cultures allow the identification of pathogens and thus contribute to quality and appropriateness of clinical care as well as efficacy of antimicrobial stewardship programs that may guide hospital resource allocation.^7^

Furthermore, follow-up blood cultures allow the identification of patients at high risk of mortality who would benefit from additional diagnostic interventions as positive follow-up cultures likely indicate more complicated infections.^8^

The performance of blood cultures has changed over decades, whereby automated blood culture systems have replaced the manual blood culture systems and enhanced their diagnostic utility, in terms of increased sensitivity and shorter time for microbial detection.^9^ Moreover, proper sampling is a prerequisite for the detection of BSIs. These steps include using alcohol-based iodine or chlorhexidine as skin antisepsis, using a butterfly needle for collection, dedicating a specialized team for sampling, providing 8–10 ml of blood inoculum in each blood culture bottle, and taking at least 2–4 sets as recommended by American Society of Microbiology/Infectious Disease Society of America.^1,10^ Despite major improvements in blood culture technology and evidence-based recommendations for sampling, blood culture contamination (BCC) remains inevitable.^10^ Clinical Laboratory Standards Institute (CLSI) recommends that the overall BCC rate should not exceed 3%,^11,12^ with evidence suggesting that rates of 1% can be achieved with proper sampling.^13^ Blood culture contaminants are usually coagulase-negative staphylococcus, *Micrococcus* spp., viridans group streptococci, *Cutibacterium*, *Corynebacterium* spp., *Clostridium perfringens* and *Bacillus* spp. that are isolated in one or two bottles within two or three sets.^11^

In this study, we determine BCC and utilization rates over a 13-year period and analyze the microbiology of the isolates. We also aim to detect any trends in BCC and blood culture utilization (BCU) before and during the COVID-19 pandemic to assess the possible impact of strict infection control practices.

## Materials and methods

### Study design and data collection

We conducted this retrospective observational study at the Saint George Hospital University Medical Center (SGHUMC), a tertiary care center in Lebanon, from January 1, 2010, to December 31, 2022. We retrieved microbiology data from the antimicrobial stewardship electronic database as well as the number of patient days (PD) and COVID-19 patient days (CPD), blood cultures, and results and number of blood cultures of patients with COVID-19 from March 2020 to March 2022. We excluded blood cultures that were obtained after more than 24 hrs of a single dose of antibiotic administration.

### Definitions

We define a contaminated blood cultures as the isolation of coagulase-negative Staphylococcus, Micrococcus species, Propionibacterium acnes, Corynebacterium species, Clostridium perfringens, or Bacillus species in one or two bottles within a four bottle set.^14^

A blood culture set refers to one aerobic and one anaerobic bottles collected from a single venipuncture site. Two sets refer to two aerobic and two anerobic bottles collected from different venipuncture sites.^11^

We define the BCU rate as the number of blood cultures processed per 1,000 PD.^15^

For COVID-19 patients, we used CPD, calculated for patients with an admitting diagnosis of COVID-19, using the “U07.1-COVID-19” ICD-10 code.

We calculated the percentage of BCC by dividing the number of contaminated blood cultures, taking into consideration one isolate per patient every 5 days, by the total number of blood cultures extracted during the same period multiplied by 100.^16^ If 3 or more positive blood cultures growing contaminants collected from patients in the absence of central lines and prosthetic materials, they were labeled as contaminated BC.

From 2020 to 2022, we categorized patients as with or without COVID-19 and calculated the percentages of BSI episodes in each category. A single BSI was defined as the growth of pathogenic organisms, such as *Staphylococcus aureus*, *Streptococcus pneumoniae*, all members of the Enterobacteriaceae family, *Pseudomonas aeruginosa*, *Acinetobacter baumannii*, and *Candida albicans* in at least one or more blood culture bottles.^2^ Each BSI episode representing the isolation of a pathogen from a blood culture was counted as 1 BSI for each 7 consecutive days, ie, duplicates were excluded.

We also recorded the location of acquisition of the BSI episode: regular floor vs ICU. All analyzed blood culture results were deduplicated (one isolate per patient).

### Statistical analysis

We determined the significance of the trends for BCU/1000 PD and BCC rates using the Mann–Kendall test and performed a time series analysis. To determine the statistical significance between the percentages of true bacteremia rates for COVID-19 and non-COVID-19 patients, we used the χ^2^ test for independence. We performed all analyses using Microsoft Excel (2015).

## Results

### General results

During the study period, we analyzed a total of 75,825 blood cultures drawn, with an average of 5,832 blood cultures per year. On average, 2 sets of blood cultures were collected per patient.

PD largely varied across the years of study, with a peak of 82,937 PD in 2015 and a low of 34,487 PD in 2022, corresponding to an average of 64,800 PD.

**Figure 1.**
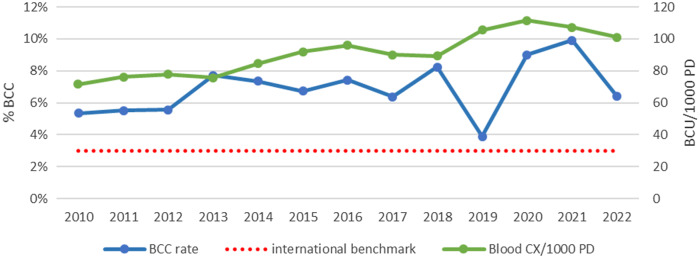
The rate of blood culture contamination (%BCC) vs blood culture utilization rates per 1000 patient days (BCU/1000 PD) from 2010 to 2022.

In this light, the lowest rate of BCU (BCU/1000 PD) in 2010 of 71.5/1000PD increases to a peak BCU of 111.5/1000 PD in 2020 with a documented upward trend in BCU/1000 PD over the years (R-squared = 0.84, p-value = 0.43) (Figure 1).

BCC rate fluctuated between 5% at the beginning of the study in 2010 and 4% in 2019, approaching the standard benchmark. However, we recorded a peak BCC of 10% in 2021, which decreased to 6% by 2022. There was no statistically significant trend over time (*P* = 0.54) (Figure 1).

### Microbiology

Skin contaminants represented 59% (5196/8780) of the total positive blood cultures and were further subdivided into their corresponding species (coagulase-negative Staphylococcus, including Staphylococcus epidermidis, Corynebacterium, diphtheroids, and gram-positive rods). We found that Staphylococcus epidermidis was the most frequent contaminant isolated throughout the years (53%–75%) followed by Corynebacterium (<10%). Diphtheroids and gram-positive rods were rarely isolated.

### Blood cultures and COVID-19

We recorded a total of 1963 blood cultures during the COVID-19 pandemic (March 2020–March 2022), of which 183 (9%) were considered contaminated. We registered 2,906 CPD in 2020, 7,682 CPD in 2021, and 994 CPD in 2022 (Table 1).

**Table 1.** Classification of blood cultures and blood culture utilization according to patients’ COVID-19 status from 2020 to 2022

	Patients with COVID-19			Patients without COVID-19		
	2020	2021	2022	2020	2021	2022
Blood cultures (=n)	426	1296	241	5870	4111	3481
Positive blood cultures (=n)	48	174	28	861	589	412
Contaminated blood cultures (=n)	37	125	21	529	408	223
Blood culture contamination rate (%)	9%	10%	8%	9%	10%	6%
Blood stream infection episodes (=n)	11	49	7	332	181	189
Blood culture utilization (BCU/1000 PD)	147	169	243	112	107	101

BCU increased steadily from 146.6/1000 CPD in 2020 to 242.5/1000 CPD in 2022. Similarly, the BCC rate was 9% in 2020, increased to 10% in 2021, and dropped back to 8% by 2022 (Table 1).

Among patients with COVID-19, contaminants represented 73% (183/250) of the total positive blood cultures. The repartition of contaminant species was similar to the general results of patients without COVID-19, with Staphylococcus epidermidis as the most common pathogen.

On the other hand, the rates of BSI episodes in patients with COVID-19 were 3%, 4%, and 3% in 2020, 2021, and 2022, respectively. Most of these BSIs, corresponding to 46/61 (75%), were acquired in the intensive care unit, reflecting the severity of their condition (Table 1). To explore whether the difference in the percentages of true bacteremia for COVID-19 patients was significantly different from those without COVID-19, we performed a χ^2^ test for independence to compare the proportions. We obtained a χ^2^ value of 5.9, which corresponds to a p-value of approximately 0.015. This indicates a statistically significant difference in the true bacteremia rates between the two groups (Table 1).

## Discussion

During our study at the SGHUMC in Lebanon, the average BCC rate was elevated at 7%, more than twice the acceptable international benchmark of 3%. This represents approximately 399 contaminated blood cultures annually, contributing to increases in the patient’s length of stay at the hospital and added cost on the healthcare system.

Moreover, it is well established that blood cultures are a major tool for BSI diagnosis.^17^ Yet, BCC remains inevitable^18^. Multiple evidence-based strategies have helped us mitigate this risk, but their role is limited. For example, skin decontamination using 2% alcoholic chlorhexidine significantly decreases the percentage of contaminated blood culture,^19^ but the contamination risk cannot be minimized to zero because 20% of the skin contaminants are located in the deeper epidermis.^20^ In addition, the use of initial specimen diversion devices reduces the contamination rate to 0.22% by diverting the first 2 mL of blood assumed to contain contaminants.^20^ Other strategies include phlebotomists training, hand hygiene, the use of sterile gloves to palpate the venipuncture site after disinfection and blood culture bottle disinfection with 70 percent isopropyl alcohol.^21^

Despite the above, we still witness high levels of BCC globally that vary between 0.6% and 12.5% among institutions^22^. Although some have reported rates as low as 0.2%.^20^, the recommended BCC benchmark is at 3%.^23^

In patients with COVID-19, we witnessed an increase in BCU rate of 185.9/1000PD, corresponding to an additional 100 blood cultures/1000CPD, which was accompanied by an elevated BCC rate of 9%. Similar rates have also been reported in other studies.^24^ During the study period involving the COVID-19 pandemic, very strict infection prevention and control methods were applied, which implies stringent hand hygiene and skin antisepsis protocols. In other words, traditional measures to mitigate risks of BCC were applied. This was met by a stable and relatively low rate of BSIs, indicating an overuse of blood cultures likely due to a sicker population but not reflective of a population where the likelihood of a BSI is elevated. Would the presence of different blood culture indications have affected the outcome?

In this retrospective study, we identified a high rate of BCC at our institution. It becomes apparent that reducing the rates of BCC starts with reducing our BCU rates by setting indications for blood cultures. The proper selection of patients with a high pretest probability of bacteremia and avoiding sampling in clinical conditions where blood cultures have lower yields than cultures from primary sources is important.^25^

Finally, this is a large retrospective, observational single center study spanning over 13 years that contributes raw, observed data. However, we are aware of its limitations that did not allow us to firmly conclude on the etiology behind BCC and its respective impact on morbidity, hospital stay, and cost. It is possible that high BCU where no specific indications guided blood culture drawing, as well as the super vigilant approach of excessive blood cultures early in the COVID-19 pandemic. We also consider the difficulty of factoring in events such as lack of skilled manpower and challenges that may affect financial and technical aspects in our healthcare system. In low-middle-income countries (LMICs), attempting to reduce these unnecessary medical and socioeconomic burdens to provide a more patient centered quality care is essential. If we take a closer look at the year 2019, we notice a rate of BCC of 4%, a value very close to the acceptable international benchmark with a more or less acceptable BCU rate of 105.6/1000 PD. Does this seem like a good balance between BCU and rate of BCC? This is possible because in 2019, we appointed a task force at SGHUMC to tackle blood culture sampling techniques including staff training sessions and the introduction of the vacutainer safety lock blood collection set (The BD Vacutainer® Safety-Lok™ blood collection set, BD medical technology). Minor changes were in progress but, unfortunately, due to several unexpected factors, we were unable to fully implement these changes. These factors included the collapsing economy in Lebanon, the Beirut port August 4, 2020, explosion, the COVID-19 pandemic, and the subsequent, unfortunate emigration of skilled healthcare professionals. This is not unusual for an LMIC. Therefore, it is crucial to adapt the issue of BCC from this angle.

Moreover, in light of limited and challenging healthcare settings, we suggest considering the possibility of establishing institution-based benchmarks or the modification of recommended guidelines and practice while catering for such challenging healthcare settings yet still ensures best safe practice. Strict adherence to aseptic techniques, revision of hospital policies and procedures, and staff training sessions are as important as the continuous surveillance of key quality performance healthcare indicators. Should BCU rate become a new key quality performance indicator? Future studies that explore the rising field of diagnostic stewardship and BCU and contamination especially in LMICs are needed to improve patient outcome and serve as boosters and support multidisciplinary antimicrobial stewardship efforts.
